# Binocular saccade coordination in reading and visual search: a developmental study in typical reader and dyslexic children

**DOI:** 10.3389/fnint.2014.00085

**Published:** 2014-10-30

**Authors:** Magali Seassau, Christophe Loic Gérard, Emmanuel Bui-Quoc, Maria Pia Bucci

**Affiliations:** ^1^e(ye)BRAIN, Ivry-sur-SeineFrance; ^2^Service de Psychopathologie de l’Enfant et de l’Adolescent, Hôpital Robert DebréParis, France; ^3^Service d’Ophtalmologie, Hôpital Robert DebréParis, France; ^4^UMR 1141 Inserm-Paris 7-Hôpital Robert DebréParis, France

**Keywords:** binocular coordination, dyslexia, reading, visual search, development

## Abstract

Studies dealing with developmental aspects of binocular eye movement behavior during reading are scarce. In this study we have explored binocular strategies during reading and visual search tasks in a large population of dyslexic and typical readers. Binocular eye movements were recorded using a video-oculography system in 43 dyslexic children (aged 8–13) and in a group of 42 age-matched typical readers. The main findings are: (i) ocular motor characteristics of dyslexic children are impaired in comparison to those reported in typical children in reading task; (ii) a developmental effect exists in reading in control children, in dyslexic children the effect of development was observed only on fixation durations; and (iii) ocular motor behavior in the visual search tasks is similar for dyslexic children and for typical readers, except for the disconjugacy during and after the saccade: dyslexic children are impaired in comparison to typical children. Data reported here confirms and expands previous studies on children’s reading. Both reading skills and binocular saccades coordination improve with age in typical readers. The atypical eye movement’s patterns observed in dyslexic children suggest a deficiency in the visual attentional processing as well as an impairment of the ocular motor saccade and vergence systems interaction.

## Introduction

Reading is a higher cognitive process depending on multiple processes: sensory perception, eye movements, linguistic and semantic capacities (Rayner et al., 2011). Furthermore, it is well known that a good control of the ocular motor system, in particular saccades, convergence and fixations, is essential for reading (Levy-Schoen and O’Regan, 1979; Seassau and Bucci, 2013). Indeed, deficits in one or more of these mechanisms could be at the origin of dyslexia. Despite intensive research on eye movements in dyslexic subjects, the origin of dyslexia is still debated, and other theories have been proposed which do not agree with an ocular motor impairment in dyslexic population (Lyon et al., 2003).

Abnormal eye movement performance observed in dyslexic children could be due to poor strategy of visual information processing, as it is not dependant on the language. A high number of regressive saccades and unstable fixation were observed by Pavlidis (1981) in Greek dyslexic children; in English dyslexic children, Rayner (1985) reported frequent saccades of smaller amplitude and longer duration fixations; in Italian dyslexic children, De Luca et al. (1999) observed frequent fixations with longer durations. More recently, slower reading speed and high number of saccades and regressions was reported in German dyslexic children (Trauzettel-Klosinski et al., 2010). Abnormal eye movements in picture searching was also reported in Chinese dyslexic children by Li et al. (2009), showing more fixations and frequent saccades of small amplitude.

Galaburda et al. (1985) was the first to show dysfunction at the level of the magnocellular system in dyslexics. Following this study, many researchers confirmed this hypothesis, showing in dyslexic population: poor binocular coordination during prolonged fixations (Stein and Fowler, 1993); visual confusion during reading (Stein and Walsh, 1997); and poor eye alignment during fixation after the saccade (Eden et al., 1994).

Confirming and extending the magnocellular hypothesis of dyslexia, impairment in visual search performance was also reported in dyslexic adults (Iles et al., 2000) with a motion coherence deficit. Even with these results, the existence of a deficiency in the magnocellular system in dyslexia is still under debate and some research does not share the hypothesis of poor visual system (Dhar et al., 2010; Skottun, 2010).

Jointly with such findings, a reduced visual attentional window size hypothesis was proposed. Bosse et al. (2007) reported that some dyslexic children have a limitation in the number of letters which can be processed in parallel. Consequently, dyslexics will make shorter saccades and frequent fixations in comparison to non dyslexic children. An fMRI study of this group (Peyrin et al., 2010) provided evidence on the role of parietal regions, particularly the left superior parietal area, in the visual attentional span and its deficiency in dyslexics. A recent study from Schneps et al. (2013) reported that young dyslexic students with reduced visual attentional span showed poor reading capabilities, suggesting a link between visual attentional spatial capabilities and dyslexia.

It is necessary to recall that the majority of research dealing with dyslexics with eye movements in reading was limited to measure movements from only one eye. Reading is, however, an activity requiring saccades and convergent eye movements. Horizontal saccades bring the eyes to successive words. For appropriate fusion of the two retinal images, the convergence angle between the two eyes needs to be well adjusted to the distance of the word. Only two studies explored binocular performance during reading in dyslexic children. Kirkby et al. (2011) reported poor binocular saccade coordination and poor fixation in dyslexic children and they suggested that reading itself could be responsible for such impairment. In contrast, a study from our group (Bucci et al., 2012) explored the quality of binocular coordination during reading and during visual search in groups of dyslexic and non dyslexic children. Disconjugacy measured during and after the saccade was significantly smaller in 10–12 year-olds than in 8–9 year-old non-dyslexic children. Furthermore, young children’s saccades were smaller in amplitude; young children fixate more often and for longer than older children. Such ocular motor behavior has been observed both while reading and searching, suggesting an immaturity of the ocular motor saccade and interaction of vergence systems.

In the present study, we have attempted to assess ocular motor performance in reading a text and visual search task in a large population of dyslexic children and compared these findings to those from typical reader children. The novelty of the present study is that we analyzed the developmental aspects of binocular eye movement behavior during reading and visual search tasks.

## Materials and methods

### Subjects

Forty three dyslexic children participated in the study. Dyslexic children were recruited from the pediatric hospital where they were referred for a complete evaluation of their dyslexic state with an extensive examination including neurological/psychological and phonological capabilities. For each child the time of reading a text, its comprehension, and the capacity of reading word/pseudowords were evaluated by using the L2MA battery (Chevrie-Muller et al., 1997). This is the standard test developed by the Centre de Psychologie appliquée de Paris, often used in France and already employed in our previous studies for selecting dyslexic population (Bucci et al., 2008, 2009). Inclusion criteria were: scores of this test beyond 2 standard deviations; a normal mean intelligence quotient (IQ, evaluated with WISC-IV; between 80 and 115). Ages of dyslexic children were comprised between 7 and 13 years (see clinical scores in Table 1). A carefully selected chronological age-matched control group (ages comprised between 7 and 13 years) of 42 typical reader children was selected. The control children had to satisfy the following criteria: no known neurological or psychiatric abnormalities, no history of reading difficulty, no visual impairment or difficulty with near vision. Also, reading capabilities within the normal range. Both the similitude test of the WISC IV assessing the verbal capability, and the matrix test of the WISC IV assessing the logic capability were performed. Normal range for both tests is 10 ± 3 (Wechsler intelligence scale for children—fourth edition, 2004). The control group was normal for verbal and for logic capabilities (see clinical scores in Table 1).

**Table 1:** **Clinical characteristic of the two groups of children examined (dyslexic and control children)**.

	**Dyslexic children**	**Control children**
**Chronological age**	10.6 (1.6)	10.7 (1.5)
**Reading age**	8.5 (1.4) *	10.3 (1.9)
**Verbal IQ**	104.8 (8)	
**Verbal Sc**.		11.7 (3.1)
**Logic IQ**	102.4 (8)	
**Logic Sc**.		11.2 (2.6)
**TNO**	58 (4.50)	47 (4.50)
**NPC**	3.55 (0.28)	4.14 (0.32)
**Heterophoria**	−2.29 (0.82)	−2.83 (0.92)
**Divergence**	10.2 (0.74) *	14.3 (0.84)
**Convergence**	28.9 (1.45) *	36.4 (1.65)

*Mean values of: chronological and reading age, IQ, binocular vision (stereoacuity test, TNO measured in seconds of arc; near point of convergence, NPC measured in cm; heterophoria at near distance measured in prism diopters; vergence fusional amplitudes (divergence and convergence) at near distance measured in prism diopters. Asterisks indicate that value is significantly different between the two groups of children (p ≤ 0.002)*.

Both typical reader and dyslexic children underwent an ophthalmologic examination of their visual sensorial and motor function (mean values showed in Table 1). The stereoacuity threshold based on disparity detection was tested with the TNO random dot test for stereoscopic depth discrimination (Netherlands Organization, Richmond Products, Boca Raton, FL, USA). To avoid the lengthy time taken by that visual test, we limited on measure convergence and divergence fusional amplitude at near distance. All children had normal binocular vision (mean value of 55 s of arc or better). Visual acuity was normal (≥20/20) for all children, dyslexic as well as typical reader. The near point of convergence was normal for both groups of children tested (mean value of 2 cm). Heterophoria at near distance (i.e., latent deviation of one eye when the other eye is covered, using the cover-uncover test) was normal for both groups of children tested (≤ exophoria of 3.5 prism D). Moreover, an evaluation of vergence fusion capability using a prisms bar was done at near distance. The divergence and convergence amplitudes were significantly different in the dyslexic group in comparison to the non dyslexic children. ANOVA showed a significant group effect for the divergence and convergence amplitudes (respectively, *F*_(1,83)_ = 13.56, *p* < 0.0005 and *F*_(1,83)_ = 11.44, *p* < 0.002). The dyslexic group had significantly smaller values of divergence and convergence compared to the typical group.

In summary, orthoptic evaluation showed a tendency of poor divergence and convergence amplitude in dyslexic children.

The investigation adhered to the principles of the Declaration of Helsinki and was approved by our Institutional Human Experimentation Committee (CPP Ile de France I, Hôpital Hotel-Dieu). Written consent was obtained from the children’s parents after an explanation of the experimental procedure.

### Ocular motor paradigms

Stimuli were presented on a PC screen of 22”, its resolution was 1920 × 1080 and the refresh rate was 60 Hz. Note that even if it is well known that intermittent illumination could affect saccade accuracy and visual assessment (Kennedy et al., 1998), such a refresh rate was sufficient to assure a normal saccade performance.

The reading and visual search tasks are similar to those used by Bucci et al. (2012) and are described below.

**Figure 1:**
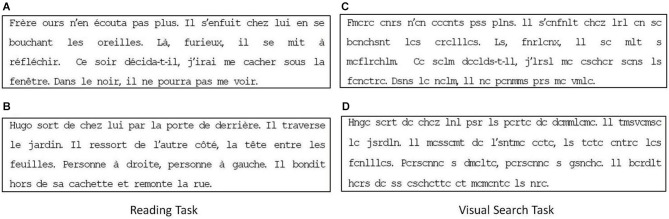
**Reading (A,B) and visual search (C,D) task respectively used for children with reading age of 8–9 and 10–13 years, respectively**.

*Reading:* A text of four lines taken from a book for children. The paragraph contained 40 words and 174 characters. The text was 29° wide and 6.4° high; mean character width was 0.5° and the text was written in black “courier” font on a white background. Text was different for the two different reading age of children examined. Figures 1A,B shows the text presented to children with a reading age of 8–9 years (extract from “*Jojo Lapin fait des farces*”, Gnid Bulton, Hachette) and that presented to children with a reading age of 10–13 years (extracted from “*Bagarres à l’école*”, Marc Cantin et Eric Gasté, Castro Cadet). Children were asked to read the text silently.

*Visual search*: The same text presented in the reading task was used for this task but vowels were replaced by consonants (see Figures 1C,D). Children were asked to count the number of “r”s occurring in the text.

In both tasks stimuli were presented without time limitation. The recording of each task stopped when the child raised one finger.

### Eye movement recordings

Eye movements were recorded with the Mobile Eyebrain Tracker (Mobile EBT^®^, e(ye)BRAIN^1^), an eye-tracking device CE marked for medical purposes. The Mobile EBT^®^ uses cameras that capture the movements of each eye independently. Recording frequency was set up to 300 Hz. The precision of this system is typically 0.5° and in controlled conditions 0.25° (see www.eye-brain.com for more details). There is no obstruction of the visual field with the recording system.

### Procedure

Children were seated on a chair in a dark room, with the head stabilized by a forehead and chin support; viewing was binocular; the viewing distance was 60 cm. Calibration was done at the beginning of eye movement recordings. The best calibration could be an haploscopic arrangement. However, it should be noted that binocular vision was normal for all children tested (see stereoacuity scores in Table 1), suggesting that they were fixating targets with both eyes. A previous study from Bucci et al. (2002) comparing typical and strabismic children without amblyopia confirmed that in the presence of normal visual acuity in both eyes either type of calibration (under monocular or binocular viewing) was valid.

During the calibration procedure, children were asked to fixate a grid of 13 points (diameter 0.5°) mapping the screen. Each calibration point required a fixation of 250 ms to be validated. A polynomial function with five parameters was used to fit the calibration data and to determine the visual angles. After the calibration procedure, the reading or visual search tasks were presented to the child. Duration of each task was kept short (lasting a couple of minutes) allowing an accurate evaluation of eye movement recordings.

### Data analysis

Calibration factors for each eye were determined from the eye positions during the calibration procedure. The software MeyeAnalysis (provided with the eye tracker, e(ye)BRAIN, France) was used to extract saccadic eye movements from the data. It automatically determines the onset and the end of each saccade by using a built-in saccade detection algorithm. The algorithm used to detect saccades is adapted from Nyström and Holmqvist (2010). The algorithm searches for velocity peaks by identifying samples where the velocity is larger than a velocity threshold (*θ* > *θ*_PT_). An iterative data-driven approach is proposed to finding a suitable threshold. The iterative algorithm is given an initial peak velocity detection threshold PT_1_, which could be in the range 100°–300°/s, but the choice is not critical as long as there are saccades, with peak velocities reaching this threshold. For all samples with velocities lower than PT_1_, the average (*μ*) and standard deviation (*σ*) are calculated. The threshold is updated as PT_*n*_ = *μ*_*n*−1_+6*σ*_*n*−1_ for each iteration. For each detected saccade peak (hose detected after the last iteration), the algorithm searches backward (from the leftmost peak saccade sample) and forward (from the rightmost peak saccade sample) in time for the saccade onset and offset. Saccade onset is defined as the first sample that goes below the saccade onset threshold and where *θ_i_*−*θ*_*i*+1_ ≥ 0. Saccadic offset is defined as the first sample that goes below the saccade offset threshold and where *θ_i_* − *θ*_*i*+1_ ≤ 0. All saccades with an amplitude superior to 1° were detected. All detected saccades were checked by the researcher and corrected/discarded if necessary.

The number and the amplitude of progressive saccades (prosaccades, from left to right) and regressive saccades (backward saccades, from right to left) and the duration of fixations between each saccade were analyzed. In both tasks (reading and visual search), binocular coordination was defined for each saccade and each fixation was recorded. For each saccade recorded in the two tasks (reading and visual search) we examined the amplitude of the conjugate [(left eye + right eye)/2], and the disconjugate components (left eye − right eye) during the saccade (see Bucci et al., 2012). The disconjugacy was measured as the change in vergence between the beginning and the end of each saccade. We also examined the disconjugate component of each post-saccadic fixation period over the period between two saccades ([(*x*_2_ − *x*_1_)_left_ − (*x*_2_ − *x*_1_)_rigth_]; where *x*_2_ = amplitude of the end of fixation and *x*_1_ = amplitude of the beginning of fixation). Given that saccade disconjugacy depends on the saccade amplitude, the values of disconjugacy during and after the saccades were presented as the ratio of the disconjugacy on the saccade amplitude (in percentage).

Statistical analysis was performed by the two-way ANOVAs using the two groups of children (dyslexics and control) as inter-subject factor and the two conditions (reading text and visual search) as within subject factor. The effect of a factor is significant when the *p*-value is below 0.05.

Then data were analyzed using different multiple linear regression models—the number of saccades, the amplitude of saccades (in degrees), the duration of fixations (in ms) and the duration of task (in seconds) for both groups. The predictor variable for each test was the participant’s age (in year and months).

## Results

### Eye movement pattern during reading and visual search (see Table 2)

#### Number of fixations

The ANOVA showed a significant group effect (*F*_(1,83)_ = 25.26, *p* < 0.0001) with a number of fixations in the control group significantly smaller than in the dyslexic group. We found also a significant effect of the task (*F*_(1,83)_ = 29.23, *p* < 0.0001), meaning that the number of fixations was larger in the visual search task with respect to the reading task. Finally, a significant interaction between group and task was also reported (*F*_(1,83)_ = 26.87, *p* < 0.0001): the control group made fewer fixations during reading than during visual search (*p* < 0.001), no difference was found between reading and visual search in dyslexic children (*p* = 0.87).

**Table 2:** **Ocular motor characteristic of the two groups of children examined (dyslexic and control children) during reading and visual search task**.

	**Reading task**		**Visual search task**	
	**Dyslexic group**	**Control group**	**Dyslexic group**	**Control group**
**Task duration (s)**	38.5 (2.6) *	13.9 (2.6)	35.2 (1.4)	31.9 (1.4)
**Nb fixations**	61 (2.52) *	38 (2.11)	61 (2.38)	57 (2.14)
**Duration of fix. (ms)**	531.1 (35.4) *	276.1 (16.4)	491.5 (17.2)	491.6 (18.6)
**Nb of progressives saccades**	50 (1.61) *	33 (1.71)	47 (1.62)	46 (1.23)
**Amplitude of progressives saccades (°)**	2.84° (0.13)* LE: 2.8 / RE: 2.8	4.03° (0.18) LE: 4.2 / RE: 3.9	3.21° (0.1) LE: 3.2 / RE: 3.2	3.05° (0.08) LE: 3.1 / RE: 2.9
**Nb of regressives saccades**	11 (1.22) *	5 (0.6)	14 (1.08) *	11 (1.08)
**Amplitude of regressives saccades (°)**	2.51° (0.13) LE: 2.5 / RE: 2.5	2.86° (0.26) LE: 2.8 / RE: 2.9	2.75° (0.10) LE: 2.7 / RE: 2.8	2.73° (0.11) LE: 2.6 / RE: 2.8
**Disconjugacy during saccades (%)**	15.4 (0.97) *	10.7 (0.70)	16.3 (1.0) *	10.9 (0.48)
**Disconjugacy after saccades (%)**	18.8 (1.4) *	9.5 (0.57)	19.5 (1.5) *	11.9 (0.45)
**Percentage of “r” counting**			94.2 (3.8)	87.7 (2.6)

*Mean values (standard error) of: task duration, number of fixations, duration of fixations, number of progressive saccades, amplitude (in °) of progressive saccades (mean amplitude and amplitude for each eye, LE = left, RE = right eye), number of regressive saccades, amplitude (in °) of regressive saccades (mean amplitude and for each eye), disconjugacy during the saccades (in %), disconjugacy after the saccades (in %), and percentage of “r” counting in the visual search. Asterisks indicate that value is significantly different between the two groups of children (p ≤ 0.01)*.

**Figure 2:**
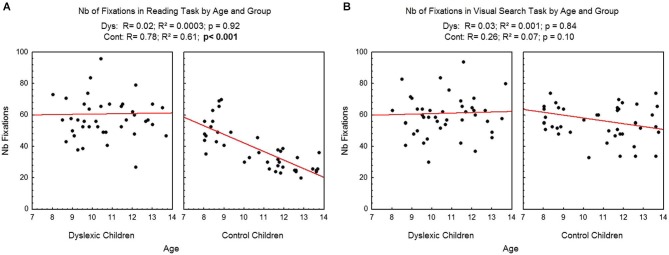
**Number of fixations during reading (A) and visual search (B) for both groups of subjects**. Lines represent the corresponding regressions.

Figure 2 shows the number of fixations assessed during reading (A) and visual search (B) as a function of age and group for each participant examined, and the regression line observed in each case. There was a significant effect of age in the reading task: the number of fixations decreased as age increased (*R*^2^ = 0.61, *p* < 0.001) only for the control children. Effect of age in reading task was not significant for dyslexic children (*R*^2^ = 0.0003). On the visual search task, age, there was no significant effect of age neither for the control children (*R*^2^ = 0.07) nor for the dyslexic children (*R*^2^ = 0.001).

#### Duration of fixations

In order to assess more information about fixations, we also measured the average duration of fixations, which is the time period between two saccades. The ANOVA showed a significant group effect (*F*_(1,83)_ = 24.14, *p* < 0.0001): the duration of fixation of the control group was significantly shorter in comparison to the dyslexic group.

We found a significant effect of the task (*F*_(1,83)_ = 18.67, *p* < 0.0001)—duration of fixations was longer in the visual search task in comparison to the reading task.

We found a significant interaction between group and task (*F*_(1,83)_ = 38.98, *p* < 0.0001); more precisely, the control group showed shorter duration of fixations in reading task in comparison to the visual search task (*p* < 0.001). No difference was found between reading and visual search in dyslexic children (*p* = 0.18).

**Figure 3:**
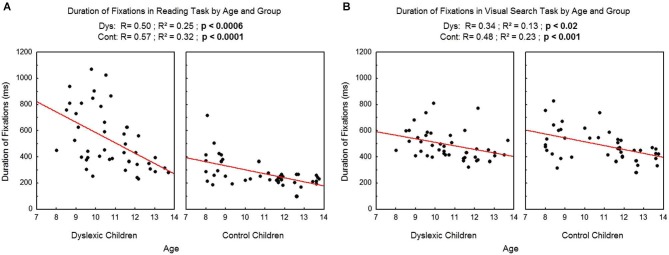
**Duration of fixations during reading (A) and during visual search (B) for the both groups of subjects**. Lines represent the corresponding regressions.

We found a significant effect of age on the duration of fixations (see Figure 3A for reading and Figure 3B for visual search task), which decreased with age in both tasks and both groups (reading: *R*^2^ = 0.25, *p* < 0.0006 and *R*^2^ = 0.32, *p* < 0.0001 respectively for dyslexic and control children; visual search: *R*^2^ = 0.13, *p* < 0.02 and *R*^2^ = 0.23, *p* < 0.001 respectively for dyslexic and control children).

#### Progressive saccade

The number of progressive saccades was significantly different between dyslexic and control groups (see Table 2). The ANOVA showed a significant group effect (*F*_(1,83)_ = 25.63, *p* < 0.0001)—dyslexic children made more progressive saccades than control children. We also found a significant task effect (*F*_(1,83)_ = 15.52, *p* < 0.001), with more progressive saccades in the visual search task than in the reading task, and a significant interaction (*F*_(1,83)_ = 4.54, *p* < 0.04) with no difference between the number of progressives saccades in reading and visual search in dyslexic children (*p* = 0.18), whereas control children made less progressive saccades in reading task compared to visual search task (*p* < 0.0001).

**Figure 4:**
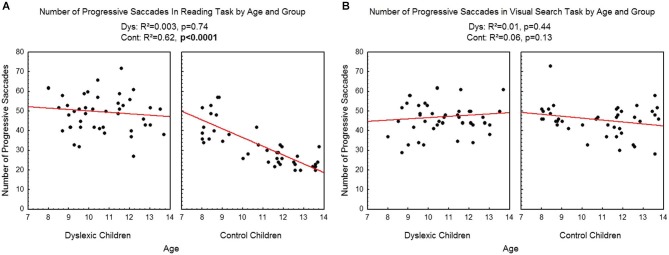
**Number of progressive saccades during reading (A) and visual search (B) for the both groups of subjects**. Lines represent the corresponding regressions.

Figure 4 shows the number of progressive saccades assessed during reading (A) and visual search (B) tasks for each participant by age and group. There was a significant effect of age on control children: the number of progressive saccades decreased with age in the reading task (*R*^2^ = 0.62, *p* < 0.0001) but not in the visual search task (*R*^2^ = 0.06, *p* = 0.13). There is no effect of age on dyslexic group neither on reading (*R*^2^ = 0.003, *p* = 0.74) nor visual search (*R*^2^ = 0.01, *p* = 0.44) tasks.

As there is no difference between amplitudes of left eye and right eye (*F*_(1,83)_ < 1), the mean amplitude of progressive saccades during reading and visual search task for each group of children is shown in Table 2. The ANOVA showed a significant group effect (*F*_(1,83)_ = 13.46, *p* < 0.0005) with smaller saccade amplitude for the dyslexic group compared to the control group. We also found a significant task effect (*F*_(1,83)_ = 6.88, *p* < 0.01), with smaller amplitude of progressive saccades in the visual search task than in the reading task.

The interaction between group and task was also significant (*F*_(1,83)_ = 34.68, *p* < 0.0001). *Post hoc* comparison showed that the amplitude of progressive saccades during reading task for the control group was significantly larger than saccades in visual search task (*p* < 0.001) and larger than in the dyslexic group (*p* < 0.0001). The amplitude of progressive saccades during reading task for the dyslexic group was significantly smaller than saccades in visual search task (*p* < 0.03). No difference was found between dyslexic and control groups in the visual search task (*p* = 0.38).

**Figure 5:**
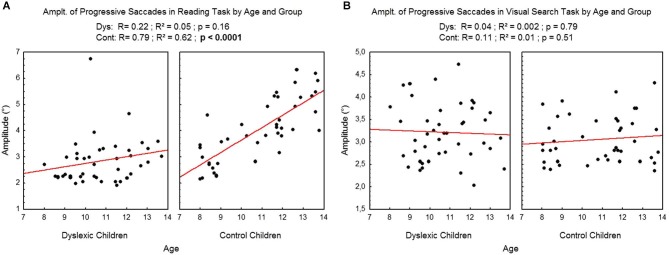
**Amplitude of progressive saccades during reading (A) and visual search (B) for the both groups of subjects**. Lines represent the corresponding regressions.

Figure 5 shows the mean amplitude of progressive saccades assessed during reading (A) and visual search (B) tasks for each participant by age and group. There was a significant effect of age on control children: the amplitude of progressive saccades increased with age in the reading task (*R*^2^ = 0.62, *p* < 0.0001) but not in the visual search task (*R*^2^ = 0.01, *p* = 0.51). There is no effect of age on dyslexic group* on reading (*R*^2^ = 0.05, *p* = 0.16) nor on visual search (*R*^2^ = 0.002, *p* = 0.79) tasks.

#### Regressive saccade

The number of regressive saccades was significantly different between the dyslexic and the control group (see Table 2). The ANOVA showed a significant group effect (*F*_(1,83)_ = 14.30, *p* < 0.0003), meaning that dyslexic children made more regressive saccades than control children. We also found a significant task effect (*F*_(1,83)_ = 33.6, *p* < 0.001), with more regressive saccades in the visual search task than in the reading task, and a significant interaction (*F*_(1,83)_ = 4.54, *p* < 0.04).

**Figure 6:**
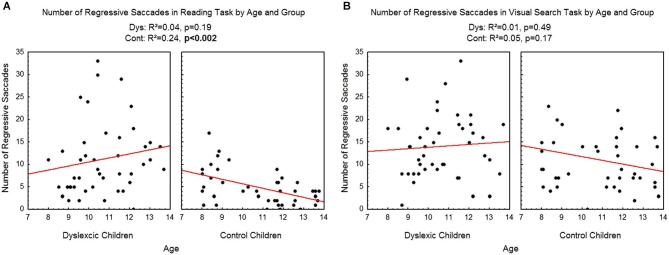
**Number of regressive saccades during reading (A) and visual search (B) for the both groups of subjects**. Lines represent the corresponding regressions.

Figure 6 shows the number of regressive saccades assessed during reading (A) and visual search (B) tasks for each participant by age and group. There was a significant effect of age on control children: the number of regressive saccades decreased with age in the reading task (*R*^2^ = 0.24, *p* < 0.002) but not in the visual search task (*R*^2^ = 0.05, *p* = 0.17). There is no effect of age on dyslexic group on reading (*R*^2^ = 0.04, *p* = 0.19) nor on visual search (*R*^2^ = 0.01, *p* = 0.49) tasks.

As there is no difference between amplitudes of left eye and right eye (*F*_(1,83)_ < 1), the mean amplitude of regressive saccades during reading and visual search task for each group of children is shown in Table 2, ANOVA showed neither a group effect (*F*_(1,78)_ < 1), nor a task effect (*F*_(1,78)_ < 1), nor a significant interaction between task and group (*F*_(1,78)_ = 2.09, *p* = 0.15). No difference was found between amplitudes of regressive saccade of dyslexic and control children in both tasks.

We did not find an effect of age on the amplitude of regressive saccades, neither in the reading task (*R*^2^ = 0.001, *p* = 0.83 and *R*^2^ = 0.04, *p* = 0.21 respectively for dyslexic and control children) nor in the visual search task (*R*^2^ = 0.02, *p* = 0.42 and *R*^2^ = 0.03, *p* = 0.31 respectively for dyslexic and control children).

### Binocular coordination during reading and visual search

#### Disconjugacy during the saccades

For the disconjugacy values reported during the saccade, the ANOVA showed a significant group effect (*F*_(1,83)_ = 34.42, *p* < 0.0001), showing that the saccades disconjugacy of the control group was significantly smaller with respect to the dyslexic group. The ANOVA did neither show a significant task effect (*F*_(1,83)_ = 1.96, *p* = 0.17) nor a significant interaction between group and task (*F*_(1,83)_ < 1).

**Figure 7:**
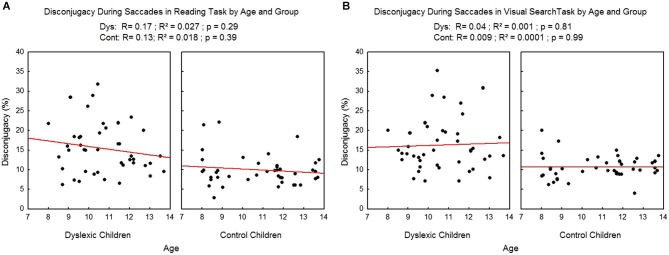
**Disconjugacy during the saccades in the reading (A) and visual search tasks (B) for both group of subjects**. Lines represent the corresponding regressions.

We found no effect of age on the disconjugacy during the saccades (see Figure 7), neither in the reading task (*R*^2^ = 0.02, *p* = 0.29 and *R*^2^ = 0.02, *p* = 0.39 respectively for dyslexic and control children) nor in the visual search task (*R*^2^ = 0.001, *p* = 0.81 and *R*^2^ = 0.009, *p* = 0.99 respectively for dyslexic and control children).

#### Disconjugacy after the saccades

Similar statistical results were reported for the values of the disconjugacy measured after the saccade. The ANOVA showed a significant group effect (*F*_(1,83)_ = 44.17, *p* < 0.0001) showing that the disconjugacy measured after the saccade of the control group was significantly smaller in comparison to the dyslexic group. The ANOVA failed to show either a significant task effect (*F*_(1,83)_ = 3.23, *p* = 0.07) or a significant interaction between group and task (*F*_(1,83)_ < 1).

**Figure 8:**
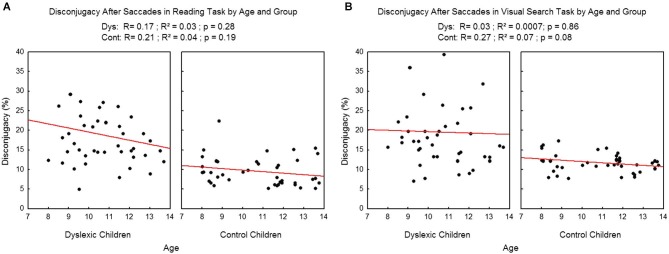
**Disconjugacy after the saccades in the reading (A) and visual search tasks (B) for both group of subjects**. Lines represent the corresponding regressions.

We found no effect of age on the disconjugacy after the saccades (see Figure 8) in the reading task (*R*^2^ = 0.03, *p* = 0.28 and *R*^2^=0.04, *p* = 0.19 respectively for dyslexic and control children). On visual search task there was no effect of age on dyslexic children (*R*^2^ = 0.0007, *p* = 0.86) and only a tendency was found in control children (*R*^2^ = 0.07, *p* = 0.08).

The performance in the visual search task was also measured (see Section Materials and Methods) by asking the child the number of “r”s read in the text. Such performance was similar in dyslexic children and control children (*F*_(1,83)_ = 2.006, *p* = 0.16). Moreover, the total task duration was no different between dyslexic and control children (*p* = 0.26) in the visual search task, whereas the duration of the reading task was significantly higher in dyslexic children in comparison to control subject (*p* < 0.0001; interaction group*task: (*F*_(1,83)_ = 30.68, *p* = 0.0001)). This data suggests that all children accomplished the visual search task in a similar way but not the reading task (See mean value in Table 1).

Finally, we explored the presence of a correlation between subjective measures of vergence clinically assessed and ocular motor measures.

In control children, disconjugacy during the saccade was correlated to convergence values measured clinically at near distance (*r* = 0.41, *p* < 0.03); in contrast, this was not the case for dyslexic children (*r* = 0.13, *p* = 0.51). The absence of such correlation in dyslexic children could be related to their large disconjugacy values reported during reading. Such finding suggests a link between saccade performance and subjective vergence capabilities (see also Bucci et al., 2011 [26]).

## Discussion

The main findings from this study are as follows: (i) during reading, ocular motor characteristics of dyslexic children are impaired in comparison to those reported in typical children; (ii) developmental effect during reading only for typical reader children. Effect of age in dyslexic children was observed only on fixation durations; (iii) different ocular motor behavior in the two tasks in dyslexic and in typical reader children; and (iv) disconjugacy during and after the saccade is larger in dyslexic children with respect to typical children.

Each of these findings is discussed.

### Ocular motor impairment of dyslexic children during reading

Many fixations, longer duration, and shorter amplitude saccades were found in dyslexic children in comparison to typical readers. These findings are in line with previous research done by recording one eye only in dyslexic children in several countries (see Section Introduction). The pattern of ocular motor impairment we reported also in this study by recording movements from both eyes could be due to an immaturity of visual attentional strategies, leading to reduced visual attentional span (which corresponds to the number of elements that can be processed in parallel) according to the study of Bosse et al. (2007). Such a limitation, leading to the higher number of fixations and longer fixation duration we reported, suggest that dyslexic children read the text analytically.

### Developmental effect on ocular motor characteristics during reading

Our findings on the number of fixations, fixation durations and amplitude saccades, during reading are in line with findings previously reported on ocular motor behavior from McConkie et al. (1991) and Blythe et al. (2006) and more recently from Seassau and Bucci (2013) showing that typical children’s reading skills develop with age. The present study showed that, with age, typical children’s reading capabilities improve and they learn to read by making larger progressive saccades, fewer regressive saccades and shorter fixations. The improvement of reading skills could be due to cortical development. Luna et al. (2008) reported that the activity of some cortical areas involved in saccadic eye movements to visual stimulus by stimulating visually-guided saccades, anti-saccades and memory-guided saccades (e.g., frontal and parietal cortex) is lower in young children than in adults and increases until adolescence. Differences in the anterior left occipito-temporal cortex was recently observed between children and adults during word processing, providing evidence of developmental course of those regions (Olulade et al., 2013). Our findings in reading deal with the developmental hypothesis. However, brain imaging studies in a large population of children during reading will be needed to further explore such an issue.

In contrast, dyslexic children’s reading skills are not influenced by age. Neither the number of fixations, nor the amplitudes of saccades nor disconjugacy are improved by age.

Further studies comparing poor readers and readers with dyslexia could be useful to better understand how eye movements reflect the difficulties that disabled readers are having understanding the text they’re reading.

### Different ocular motor behavior in the two tasks in dyslexic and in typical reader children

The reading and the visual search tasks made different demands on visuo-perceptual, attentional and spatial processing. Consequently one could expect to observe different ocular motor behavior in these two tasks. Typical reader children showed significant differences between the two tasks only on the number of fixations, fixation durations and amplitude of saccades. Note that the pattern of fixation is different in the two tasks because they correspond to different cognitive demands in the case of well-reading children. In the visual search task, the child is required to identify and count a single target; he has to see all the letters in order to adequately perform the task. In contrast, in the reading task the child can skip letters because the linguistic processing is well developed. Consequently, reading is easier than visual searching for typical reader children.

In contrast, dyslexic children display similar ocular motor behaviors in both tasks. According to Prado et al. (2007) a reduced visual attentional span could have a similar impact on reading and on visual search, because visual attentional demand is similar in the two tasks. In dyslexic children, as reading capabilities are not well structured, reading and visual search tasks had similar demands in visuo-perceptual, attention and spatial processing. We suggest that dyslexic children perform both tasks in a similar way. In contrast, typical readers, who have better developed reading skills, accomplish both tasks differently, which is reflected in their ocular motor behavior.

Finally, it should be pointed out that the two tasks did not show any difference with regards to the binocular coordination of saccades, neither in typical nor dyslexic readers. Previously, we have already showed that the quality of binocular coordination during and after the saccades does not depend on the stimulus used (single word reading; fixation of LEDs or text reading; Bucci and Kapoula, 2006; Bucci et al., 2012). These results are also in line with the study of Jainta and Kapoula (2011) comparing binocular saccade coordination during reading and free exploration of painting. The present data brings new evidence on the quality of binocular coordination by showing that reading texts do not interfere with, and contrasts Heller and Radach (1999) and Kirkby et al. (2011) reports, suggesting that reading itself induces impairment in the binocular saccade control and fixation instability.

Note, however, that further studies exploring binocular coordination on linguistic and non linguistic stimuli could be useful to better understand how binocular coordination could be influenced or not by the type of visual stimuli.

### Large disconjugacy during and after the saccades in dyslexic children

The poor quality of binocular coordination in dyslexic children, during and after the saccades, suggests an impairment of ocular motor learning mechanisms, at central/cortical level responsible for saccade yoking. In dyslexic children the clinically assessed limited vergence capabilities (see Table 1) could be responsible for such a deficient interaction between saccadic and vergence movements and thus lead to disconjugate saccades.

According to Lewis et al. (1995), we can hypothesize that the fine control of binocular saccade coordination is based on an efficient relationship between the motor command of the saccades and the vergence subsystems at the premotor level. This hypothesis has been tested in different types of child populations showing poor vergence fusional capabilities (i.e., dyslexic children, children with strabismus and children with vergence insufficiency) (Bucci et al., 2012; Gaertner et al., 2013; Lions et al., 2013). Note that reading is an activity done at near distance. In order to adjust the visual axes of both eyes at the distance of the word, a correct convergence command strictly linked with the saccade command is needed for appropriate fusion of the two retinal images. All child populations with poor vergence capabilities (as those previously cited) showed poor binocular saccade control. This hypothesis, however, needs further exploration.

We could make the hypothesis that vergence training could help dyslexic children to improve the quality of their saccade coordination. This hypothesis, however, needs further exploration.

Finally, a fine binocular coordination of saccades could involve the magnocellular network and also the cerebellum according to the study of Nicolson et al. (1999). However, according to Iles et al. (2000), deficits in the magnocellular network involving the parietal cortex could be related to poor visuo-attentional capabilities already reported in dyslexic children. Further studies by combining neuroimaging techniques and visuo-attentional tasks will be necessary to test the different hypothesis on the origin of dyslexia.

## Conclusion and future directions

Deficits in ocular motor behavior reported in dyslexic children seem to be associated to the precise controlled interaction between the saccade and the vergence systems. This ocular motor deficit could be in relationship with the clinical assessment, showing poor fusional vergence capabilities in dyslexic children.

We believe that orthoptic vergence training, together with specific visual attentional training and reading tasks, could be useful tools for dyslexic children to improve visual attentional span and vergence capabilities as well as saccade yoking.

## Funding

The authors have no support or funding to report.

## Conflict of interest statement

Magali Seassau declares work for the e(ye)BRAIN company and author as co-inventor of a patent (B110332FRA, 2011) for the detection of an oculomotor abnormality (parameter for binocular coordination) in a dyslexic patient by means of a visual search test, which is used in this study. Other authors have declared that no competing interests exist.

## Footnotes

^1^www.eye-brain.com
